# Occupational health nurse involvement and Bright 500 certification among Japanese small- and medium-sized enterprises: a repeated cross-sectional analysis of 4 annual health and productivity management survey waves

**DOI:** 10.1093/joccuh/uiag031

**Published:** 2026-06-22

**Authors:** Satoru Kanamori, Kumi Sugimoto, Satoshi Miyata, Junko Nakatani, Tomohisa Nagata

**Affiliations:** Graduate School of Public Health, Teikyo University, 2-11-1 Kaga, Itabashi-ku, Tokyo 173-8605, Japan; Department of Preventive Medicine and Public Health, Tokyo Medical University, Tokyo, Japan; Graduate School of Public Health, Teikyo University, 2-11-1 Kaga, Itabashi-ku, Tokyo 173-8605, Japan; PONO Inc, Tokyo, Japan; Graduate School of Public Health, Teikyo University, 2-11-1 Kaga, Itabashi-ku, Tokyo 173-8605, Japan; School of Health Sciences, University of Occupational and Environmental Health, Kitakyushu, Japan; Department of Occupational Health Practice and Management, Institute of Industrial Ecological Sciences, University of Occupational and Environmental Health, Kitakyushu, Japan

**Keywords:** occupational health nursing, occupational health services, health promotion, workplace, small business, Japan

## Abstract

**Objectives:**

To investigate the association between occupational health nurse (OHN) involvement and Bright 500 certification, an indicator of high-performing health and productivity management (HPM), among Japanese small- and medium-sized enterprises (SMEs) using multi-year survey data.

**Methods:**

This observational study used secondary data from the 2021-2024 HPM Survey. Each annual response was analyzed as a corporation-year observation, and within-corporation correlation was handled with a random intercept for corporations. OHN involvement was defined as the appointment of public health nurses and/or nurses as health promotion officers. Bright 500 certification, obtained from publicly available certification records, was used as an indicator of more advanced HPM. The association was examined using a mixed-effects logistic regression model.

**Results:**

The analytical sample comprised 12 847 (2021), 14 401 (2022), 17 316 (2023), and 20 267 (2024) SMEs. OHN involvement increased from 5.1% (2021) to 5.8% (2024). Bright 500 certification was consistently higher in corporations with OHN involvement (10.8% in 2021, 8.4% in 2024) than in those without (2%-3% across years). After adjustment for survey year, region, insurer category, industry type, employee size, internal and external dissemination of HPM initiatives, and occupational physician involvement, OHN involvement was independently associated with certification (odds ratio: 2.35; 95% CI, 1.21-4.54; *P* = .011).

**Conclusions:**

Across the 4 survey waves, OHN involvement was positively associated with Bright 500 certification among SMEs. These findings suggest that OHNs may be relevant to advanced HPM implementation in this setting. Future studies should capture OHN staffing arrangements, intensity and roles, and initiative timing to clarify mechanisms and potential causality.

## Teaser

**Key points**
 

**What is already known on this topic**:
  - The well-being of workers is a major international priority amid workforce aging and the increasing burden of chronic and mental health conditions. Employer investment in employee health is linked to productivity, work engagement, and organizational sustainability; Japan’s health and productivity management (HPM) strategy embeds this goal in corporate strategies. However, small- and medium-sized enterprises (SMEs) face structural constraints, and occupational health activities are often limited to legal compliance. Occupational health nurses (OHNs) can bridge management and frontline practice; nonetheless, evidence linking OHN involvement to HPM maturity has been largely cross-sectional.
**What this study adds**:
  - Across the 4 waves (2021–2024) of the HPM Survey, OHN involvement was consistently more common among SMEs with Bright 500 certification, a proxy for more advanced HPM. In a mixed-effects logistic regression model adjusted for organizational and contextual factors (including occupational physician involvement), OHN involvement was independently associated with Bright 500 certification (odds ratio: 2.35; 95% CI, 1.21-4.54).
**How this study might affect research, practice, or policy**:
  - OHN involvement was associated with Bright 500 certification among Japanese SMEs after adjustment for organizational and contextual factors, suggesting that OHNs may be a workforce element relevant to HPM advancement in this setting. However, because the present analysis did not assess specific OHN activities, staffing arrangements, or organizational role configurations, further research directly measuring these process variables is needed to clarify the mechanisms underlying this association and to inform specific implications for SME HPM design and policy.

## 1. Introduction

Workforce aging, along with the increasing burden of chronic diseases and mental health problems, has raised the well-being of workers and comprehensive health promotion in the workplace as major international policy and management priorities. Workers spend a substantial proportion of their time at work, making the workplace an effective setting for interventions that can simultaneously affect their physical, mental, and social health.^1^^,^^2^ Corporate investment in employee health is associated with improved health outcomes, higher productivity, stronger work engagement, and enhanced corporate sustainability.^3^ Thus, health initiatives are increasingly framed as strategic investments in human capital.^1^

Within this broader international movement, Japan’s health and productivity management (HPM) strategy has integrated workplace health initiatives into corporate strategies, with worker well-being serving as a key outcome. HPM is a management approach that positions employee health as a key managerial resource and seeks to enhance corporate productivity and value through organized and strategic efforts to maintain and promote health; in Japan, this approach has been institutionally developed under strong government leadership.^4^^,^^5^ Consistent with international concepts of workplace health promotion, HPM can be regarded as an implementation model that views employee health as human capital while aligning it with sustained organizational growth.^6^^,^^7^ Implementation constraints related to enterprise size, particularly among small- and medium-sized enterprises (SMEs), have been repeatedly noted.

According to the International Labour Organization, SMEs often lack institutional, human, and financial resources compared with large enterprises, resulting in structural constraints on health and safety promotion.^8^ Access to occupational health professionals such as occupational physicians and occupational health nurses (OHNs) is often constrained, and health initiatives may be insufficiently embedded in executive decision-making and routine organizational management. Comparative research between Japan and the Netherlands suggests that occupational physician activities in Japan’s small workplaces involve shorter activity times and focus primarily on legal compliance, and that occupational physicians in both countries emphasize the importance of employer education and broader organizational support, including health promotion, to sustain occupational health activities in SMEs.^9^^,^^10^ In Japan, occupational physicians are statutorily required only at workplaces with 50 or more regular employees and typically engage on a limited part-time basis, whereas OHNs are not subject to such size-based mandates. Therefore, voluntary OHN appointment in SMEs may reflect not only an orientation toward sustained, process-oriented HPM beyond minimum compliance but also organizational resources and support systems.

In this context, OHNs have been recognized in Japan as potentially important workforce members for strengthening HPM in SMEs. Core competencies of OHNs include strategic planning, interprofessional coordination, support for client growth, and team empowerment.^11^ Their frequent workplace engagement positions them as health professionals who support day-to-day behavioral changes and, critically, translate health priorities into feasible actions for managers and frontline staff. A previous Japanese study revealed that workplaces with OHNs had higher implementation rates of health promotion activities across multiple domains, including nutrition, exercise, smoking cessation, alcohol consumption, and mental health.^12^ Studies on Japanese SMEs further described facilitation models in which OHNs empowered both employers and employees by using practical tools such as action checklists, enabling locally owned initiatives despite resource constraints.^13^ Furthermore, previous Japanese studies showed that education and support for managers by occupational health staff were associated with higher perceived organizational support among managers,^14^ and that professional participation in executive-level meetings was also associated with higher overall scores and higher “evaluation/improvement” ratings in the HPM Survey, where this rating reflected the quality of continuous improvement processes.^15^ These patterns are evident in Japanese small- and medium-sized workplaces, and continuous external organizational support by independent OHNs and others reportedly improves the implementers’ literacy and autonomy while promoting changes in executives’ awareness and behaviors that contribute to sustained implementation.^16^

Despite this growing body of research, important gaps remain. Several investigations of OHN involvement and HPM outcomes have been based on single-year cross-sectional data, making it difficult to determine whether the observed associations are consistent across multiple years. In addition, nationwide data on OHN involvement among Japanese SMEs remain scarce, limiting benchmarking across studies. Moreover, outcome measures capturing organizational maturity and continuous improvements remain limited. Against this backdrop, the Bright 500 certification can be considered a practical indicator because it not only assesses the sustained implementation of workplace health initiatives and related improvement processes under external evaluation but also reflects factors such as organizational capacity, industry context, and engagement with the application process, which underscores the value of evaluating the association using multiple annual waves rather than a single year.

The present study aimed to investigate the association between OHN involvement and Bright 500 certification using data derived from the 4 annual waves of the HPM Survey in Japan, applying a mixed-effects model that accounts for repeated participation by the same corporation across waves. In this study, Bright 500 certification, which is granted to the top 500 SMEs under the program and is therefore considered a practical indicator of high-performing organizations among SMEs,^17^ was used as a proxy indicator of organizational attainment and maturity in HPM.

## 2. Methods

### 2.1 Study design and study population

This observational study analyzed secondary data from the HPM Survey conducted in Japan as part of the HPM initiative promoted by the Ministry of Economy, Trade, and Industry (METI). The HPM Survey is administered nationwide annually to evaluate the status of HPM initiatives among corporations.^18^ For this study, corporate-level response data from this survey, which are managed by the METI, were provided for research purposes following prescribed procedures. SMEs that responded to the survey at 4 time points from 2021 to 2024 were included in the analysis.

Although each annual survey is cross-sectional, some corporations have participated repeatedly over multiple years. Thus, the response from each corporation in each year was defined as 1 observation unit (corporation-year data) and treated as repeated-measures data. This approach enabled analyses that accounted for temporal correlations within corporations.

### 2.2 Exposure

The exposure of interest was OHN involvement in a corporation. Exposure status was determined based on survey responses. Corporations that assigned health promotion officers at all business sites were identified. OHN involvement was defined as the appointment of public health nurses and/or nurses as health promotion officers, whereas all other corporations were classified as having no OHN involvement. Exposure was assessed separately for each survey year and operationalized at the corporation-year level.

### 2.3 Outcome

The primary outcome was Bright 500 certification in SMEs. Bright 500 certification is a designation awarded to the top 500 small- and medium-sized corporations with high HPM initiative evaluation scores based on the HPM Survey and application process administered by the METI.^17^ Outcome data were derived from annual survey results and publicly available certification records. Information on Bright 500 certification status (defined as a binary variable [certified vs uncertified] for each corporation-year observation) was extracted from the certification results released by the METI and the HPM Organization Certification Office.^18^ These data were downloaded on June 10, 2025, and used for analysis.

### 2.4 Other variables

Several covariates based on previous studies^12^^,^^14^^,^^15^ and survey structure were included to investigate the association between OHN involvement and outcomes. Region was categorized into 6 areas according to the prefecture of the corporate headquarters. The insurer category was originally classified into 4 types in the survey: the Japan Health Insurance Association (Kyokai Kenpo), health insurance societies, National Health Insurance (NHI) associations (eg, construction-related NHI), and mutual aid associations. For analysis, these categories were reclassified into 2 groups according to their institutional characteristics: the Japan Health Insurance Association and NHI were combined into a single category comprising community-based and small employer insurers, whereas society-managed health insurance and mutual aid associations/others were combined into another category comprising large employer and public sector insurers. The economic sector was classified into primary/secondary sectors and tertiary/other sectors. The number of employees was categorized as ≤49, 50-99, or ≥100. Internal dissemination of HPM initiatives was operationalized as a continuous variable representing the total number of internal HPM communication practices implemented by the corporation; this variable was constructed by summing affirmative (“yes”) responses to 7 survey items related to internal dissemination, such as direct notifications to employees, circulation of written materials, and postings on bulletin boards or internal intranet systems. External dissemination of HPM initiatives was assessed as the number of external HPM communication practices, which included 6 survey items such as posts at office entrances and disclosures of HPM initiatives on the corporation’s official website. Occupational physician involvement was treated as a binary variable using criteria analogous to those applied to OHNs. All covariates were assessed annually and analyzed at the corporation-year level.

### 2.5 Statistical analysis

The association between OHN involvement and Bright 500 certification was analyzed using a mixed-effects logistic regression model. The dependent variable was Bright 500 certification status, whereas the primary independent variable was OHN involvement. Corporation-year observations constituted the analytical units, and corporations were included as random intercepts to account for correlations between repeated observations within the same corporation. The model was adjusted for the survey year, region, insurer category, industry type, number of employees, internal and external dissemination of HPM initiatives, and occupational physician involvement. The survey year was included as a fixed effect to account for temporal changes in institutional and social contexts. The results were reported as odds ratios (ORs) with 95% CIs. All analyses were performed using R version 4.5.2 (R Foundation for Statistical Computing, Vienna, Austria),^19^ with the 2-sided significance level set at 5%.

### 2.6 Ethical considerations

The data used in this study were existing corporate-level survey data managed by the METI and did not include personal information. Data were analyzed in an aggregated form at the corporate level, and no information allowing the identification of individuals was included.

Permission to use individual survey response data was obtained through a formal application to the METI, which specified the academic purpose and handling of research outputs. In accordance with the Japanese Ethical Guidelines for Medical and Biological Research Involving Human Subjects,^20^ this study using existing data without personal information was exempt from ethical review; informed consent and ethics committee approval were not required. Nonetheless, careful attention was paid to protecting corporate information and implementing appropriate data management strategies.

## 3. Results

SMEs were analyzed using repeated cross-sectional data collected at 4 time points between 2021 and 2024. The analytical sample comprised 12 847 SMEs in 2021, 14 401 SMEs in 2022, 17 316 SMEs in 2023, and 20 267 SMEs in 2024. The percentage of corporations with OHN involvement increased over time, from 5.1% in 2021 to 5.8% in 2024.

The OHN involvement group included a higher proportion of corporations with a larger employee size and a higher proportion enrolled in large employer and public sector insurers than the no-OHN involvement group. The OHN involvement group also showed a higher rate of occupational physician appointments and a higher mean number of internal and external HPM communications. Bright 500 certification was consistently more common among corporations with OHN involvement (10.8% in 2021 and 8.4% in 2024) than among those without OHN involvement (approximately 2%-3% across the years). Additional corporate characteristics according to OHN involvement by survey year are summarized in Table 1. The distributions of OHN involvement and other explanatory variables according to Bright 500 certification status by survey year are presented in Table S1.

**Table 1:** Characteristics of SMEs by OHN involvement (2021-2024).

Year	2021 (*n* = 12 847)			2022 (*n* = 14 401)			2023 (*n* = 17 316)			2024 (*n* = 20 267)		
	OHN involvement			OHN involvement			OHN involvement			OHN involvement		
	Total	Yes	No	Total	Yes	No	Total	Yes	No	Total	Yes	No
**Total**	12 847 (100.0)	655 (100.0)	12 192 (100.0)	14 401 (100.0)	751 (100.0)	13 650 (100.0)	17 316 (100.0)	932 (100.0)	16 384 (100.0)	20 267 (100.0)	1182 (100.0)	19 085 (100.0)
**Region**												
**Hokkaido–Tohoku**	1624 (12.6)	69 (10.5)	1555 (12.8)	1738 (12.1)	68 (9.1)	1670 (12.2)	2114 (12.2)	92 (9.9)	2022 (12.3)	2534 (12.5)	116 (9.8)	2418 (12.7)
**Kanto**	2032 (15.8)	177 (27.0)	1855 (15.2)	2422 (16.8)	199 (26.5)	2223 (16.3)	3127 (18.1)	245 (26.)3	2882 (17.6)	3734 (18.4)	303 (25.6)	3431 (18.0)
**Chubu**	3134 (24.4)	169 (25.8)	2965 (24.3)	3644 (25.3)	188 (25.0)	3456 (25.3)	4317 (24.9)	235 (25.2)	4082 (24.9)	5164 (25.5)	298 (25.2)	4866 (25.5)
**Kinki**	3371 (26.2)	115 (17.6)	3256 (26.7)	3568 (24.8)	132 (17.6)	3436 (25.2)	4131 (23.9)	160 (17.2)	3971 (24.2)	4714 (23.3)	220 (18.6)	4494 (23.5)
**Chugoku–Shikoku**	1615 (12.6)	54 (8.2)	1561 (12.8)	1807 (12.5)	77 (10.3)	1730 (12.7)	2068 (11.9)	89 (9.5)	1979 (12.1)	2331 (11.5)	106 (9.0)	2225 (11.7)
**Kyushu**	1068 (8.3)	71 (10.8)	997 (8.2)	1221 (8.5)	87 (11.6)	1134 (8.3)	1553 (9.0)	110 (11.)8	1443 (8.8)	1790 (8.8)	139 (11.8)	1651 (8.7)
**Health insurer**												
**Large employer and public sector insurers**	1834 (14.3)	221 (33.7)	1613 (13.2)	2267 (15.7)	258 (34.4)	2009 (14.7)	2842 (16.4)	309 (33.2)	2533 (15.5)	3474 (17.1)	388 (32.8)	3086 (16.2)
**Community-based and small employer insurers**	11 006 (85.7)	434 (66.3	10 572 (86.7)	12 129 (84.2)	493 (65.6)	11 636 (85.2)	14 471 (83.6)	623 (66.8)	13 848 (84.5)	16 793 (82.9)	794 (67.2)	15 999 (83.8)
**Economic sector**												
**Primary and secondary sectors**	5392 (42.0)	195 (29.8	5197 (42.6)	6380 (44.3)	249 (33.2)	6131 (44.9)	7801 (45.1)	307 (32.9)	7494 (45.7)	9324 (46.0)	392 (33.2)	8932 (46.8)
**Tertiary and other sectors**	7455 (58.0)	460 (70.2	6995 (57.4)	8021 (55.7)	502 (66.8)	7519 (55.1)	9515 (54.9)	625 (67.1)	8890 (54.3)	10 943 (54.0)	790 (66.8)	10 153 (53.2)
**Number of employees**												
**≤49**	8095 (63.0)	310 (47.3	7785 (63.9)	8771 (60.9)	334 (44.5)	8437 (61.8)	10 468 (60.5)	418 (44.8)	10 050 (61.3)	12 008 (59.2)	510 (43.1)	11 498 (60.2)
**50-99**	2221 (17.3)	142 (21.7	2079 (17.1)	2586 (18.0)	162 (21.6)	2424 (17.8)	3109 (18.0)	201 (21.6)	2908 (17.7)	3730 (18.4)	263 (22.3)	3467 (18.2)
**≥100**	2531 (19.7)	203 (31.0	2328 (19.1)	3044 (21.1)	255 (34.0)	2789 (20.4)	3739 (21.6)	313 (33.6)	3426 (20.9)	4528 (22.3)	409 (34.6)	4119 (21.6)
**Number of internal HPM communications, mean (SD)**	2.87 (1.55)	3.56 (1.75	2.83 (1.53)	3.00 (1.58)	3.71 (1.80)	2.96 (1.56)	2.97 (1.63)	3.72 (1.79)	2.92 (1.61)	2.98 (1.63)	3.68 (1.81)	2.93 (1.61)
**Number of external HPM communications, mean (SD)**	2.12 (1.14)	2.52 (1.38	2.09 (1.12)	2.19 (1.20)	2.64 (1.38)	2.17 (1.18)	2.19 (1.22)	2.65 (1.42)	2.17 (1.21)	2.21 (1.24)	2.67 (1.42)	2.18 (1.23)
**Occupational physician involvement**												
**Yes**	846 (6.6)	248 (37.9	598 (4.9)	1050 (7.3)	301 (40.1)	749 (5.5)	1354 (7.8)	379 (40.7)	975 (6.0)	1695 (8.4)	479 (40.5)	1216 (6.4)
**Bright 500 certification**												
**Yes**	502 (3.9)	71 (10.8	431 (3.5)	499 (3.5)	91 (12.1)	408 (3.0)	495 (2.9)	87 (9.3)	408 (2.5)	499 (2.5)	99 (8.4)	400 (2.1)

Abbreviations: HPM, health and productivity management; OHN, occupational health nurse; SME, small- and medium-sized enterprise.

Values are presented as *n* (%) unless otherwise indicated.

The association between OHN involvement and Bright 500 certification was examined using a mixed-effects logistic regression model adjusted for the survey year, region, insurer category, industry type, number of employees, internal and external dissemination of HPM initiatives, and occupational physician involvement (Table 2). OHN involvement was associated with significantly higher odds of Bright 500 certification (OR: 2.35; 95% CI, 1.21-4.54; *P* = .011).

**Table 2:** Mixed-effects logistic regression model for Bright 500 certification among SMEs, 2021-2024.

	OR (95% CI)	*P*
**Year (continuous)**	0.62 (0.56-0.68)	<.001
**Region (ref: Kanto)**		
**Hokkaido-Tohoku**	0.19 (0.04-0.84)	.029
**Chubu**	0.64 (0.20-2.02)	.449
**Kinki**	0.17 (0.05-0.63)	.008
**Chugoku-Shikoku**	0.21 (0.04-1.04)	.056
**Kyushu**	0.61 (0.14-2.65)	.512
**Health insurer (ref: Large employer and public sector insurers)**	0.64 (0.23-1.80)	.396
**Economic sector (ref: Tertiary and other sectors)**	0.82 (0.36-1.87)	.641
**Number of employees (ref: ≤49)**		
** 50-99**	0.47 (0.24-0.93)	.031
** ≥100**	0.65 (0.29-1.45)	.290
**Number of internal HPM communications (continuous)**	1.77 (1.53-2.06)	<.001
**Number of external HPM communications (continuous)**	2.50 (2.11-2.96)	<.001
**Occupational physician (ref: No)**	1.50 (0.93-2.43)	.097
**Occupational health nurse (ref: No)**	2.35 (1.21-4.54)	.011

Abbreviations: HPM, health and productivity management; OR, odds ratio.

A mixed-effects logistic regression model was used with random intercepts for companies to account for within-company correlation across survey years. Year and all covariates were included as fixed effects.

## 4. Discussion

This study investigated the association between OHN involvement and Bright 500 certification among SMEs using data from the 2021-2024 HPM Survey (a 4-wave repeated cross-sectional dataset). The proportion of corporations with OHN involvement showed a modest year-on-year increase, and Bright 500 certification was consistently more frequent among corporations with OHN involvement than among those without OHN involvement. OHN involvement was independently associated with Bright 500 certification in a mixed-effects logistic regression model adjusted for survey year, region, insurer category, industry type, number of employees, internal and external dissemination of HPM initiatives, and occupational physician involvement. Corporations with OHN involvement exhibited approximately 2.35-fold higher odds of Bright 500 certification (OR: 2.35; 95% CI, 1.21-4.54: *P* = .011).

In the present study, OHN involvement was associated with higher adjusted odds of obtaining Bright 500 certification, consistent with findings from previous studies reporting that OHNs can facilitate the implementation of workplace health promotion activities. For instance, a previous report showed that workplaces with OHNs implemented a broader range of health promotion activities spanning nutrition, physical activity, smoking cessation, alcohol consumption, and mental health.^12^ A cross-sectional study analyzing 2021 application data for the HPM Outstanding Corporation (SME category) reported that the acquisition of Bright 500 certification was associated with occupational health activities, including the role and qualifications of the staff in charge, provision of specific health guidance, and post-checkup counseling; notably, “collaboration with occupational health staff” was strongly associated with Bright 500 certification.^21^ While much of the existing evidence is based on single-year cross-sectional analyses, the present study provides a complementary perspective by using multi-year corporation-year data from 4 survey waves. Specifically, the analysis adjusted for survey year and accounted for within-corporation correlation among repeated observations. In this way, our findings align with the results of prior studies while reinforcing the robustness of the association between OHN involvement and Bright 500 certification using data spanning multiple years.

Several mechanisms have been proposed in the prior literature that could plausibly explain a positive association between OHN involvement and Bright 500 certification, although the present study did not directly examine these mechanisms. Prior studies have suggested that management discussions of HPM and the involvement of occupational health professionals in these discussions are associated with HPM evaluation (ie, outcomes of workplace health promotion programs),^15^ and that professional participation in decision-making and progress monitoring may translate into stronger implementation and iterative improvement. Other prospective evidence has suggested that occupational health staff involvement may enhance perceived organizational support among managers,^14^ which in turn could strengthen managerial involvement and thereby facilitate HPM implementation. These prior findings collectively suggest that OHN involvement may support organizational processes central to “mature HPM,” such as connecting management, supervisors, and external resources and implementing plan–do–check–act cycles. However, the present study analyzed only Bright 500 certification as an outcome and did not include process variables such as the implementation status of specific health promotion activities, the content of management discussions, or perceived organizational support. The mechanisms outlined above therefore remain hypothetical in the context of our data, and future studies incorporating direct measures of these process variables are needed to clarify the pathways through which OHN involvement may contribute to mature HPM.

As this was an observational analysis, the findings do not establish a causal effect of OHN involvement on Bright 500 certification. Nonetheless, considering the observed association and prior literature, an implementation-relevant question for SMEs is how to position OHNs with respect to their delivery arrangements and functional roles. Even when full-time OHN placement is impractical, OHNs affiliated with health insurers have been reported to function as external supporters (facilitators) who provide repeated ongoing visits across multiple workplaces and employ tools such as action checklists to empower employers and employees, thereby supporting the implementation of workplace health promotion activities.^13^ Such facilitative roles are consistent with the competencies expected of OHNs, including strategic planning, interprofessional coordination, support for client growth, and team empowerment.^11^ However, specific action recommendations cannot be derived from these data alone because specific OHN activities, staffing arrangements, and organizational role configurations were not assessed. These issues should be examined in future studies.

This study had some limitations. First, this observational study relied on secondary HPM Survey data and adopted a repeated cross-sectional approach that treated each annual response as corporation-year data; thus, causal interpretation was limited. Reverse causality is possible if corporations recruit or intensify OHN involvement while pursuing certification, and residual confounding by unmeasured factors (eg, management commitment and available resources for HPM) cannot be fully excluded. Although a mixed-effects model with a corporate random intercept partially accounted for within-corporation correlation and time-invariant unmeasured characteristics, it could not adequately address time-varying unmeasured factors or fully identify the temporal sequences of initiatives. Second, OHN involvement was defined using survey items on the appointment of “health promotion officers” and their qualifications (public health nurses/nurses), precluding assessment of staffing arrangements (dedicated vs concurrent roles, full-time vs part-time status, outsourcing) and the intensity and scope of OHN involvement; therefore, exposure misclassification is possible. Related covariates such as occupational physician involvement and internal/external dissemination were also self-reported, raising the possibility of reporting bias. Third, because the Bright 500 certification is a relative designation awarded to the top 500 corporations, certification rates and association estimates may vary with annual application patterns and changes in evaluation frameworks. Fourth, because participation in the HPM Survey is voluntary, corporations with a stronger interest in or more advanced HPM initiatives may have been more likely to respond. If such a selection is associated with both OHN involvement and Bright 500 certification, the estimated association may be biased in either direction.

## 5. Conclusions

This study investigated the association between OHN involvement and Bright 500 certification among SMEs participating in the 2021-2024 HPM Survey from a multi-year repeated cross-sectional perspective. OHN involvement was independently and positively associated with Bright 500 certification in a mixed-effects logistic regression model adjusted for the survey year, region, insurer category, industry type, number of employees, internal and external dissemination of HPM initiatives, and occupational physician involvement. Corporations with OHN involvement had significantly higher odds of certification than those without OHN involvement. These findings suggest that OHN involvement may be associated with more advanced HPM efforts among SMEs, as indicated by Bright 500 certification. Future research should incorporate OHN staffing arrangements, intensity and roles of OHN involvement, and temporal ordering of initiatives to clarify the underlying mechanisms and further evaluate potential causal relationships.

## Supplementary Material

Supplementary_Table_1_uiag031

## Data Availability

The corporate-level survey data used in this study were provided by the Ministry of Economy, Trade, and Industry (METI) under an approved application process. These data are not publicly accessible; however, they may be obtained from METI upon reasonable request and in accordance with data-sharing procedures and permissions.
